# Light and Food: Body Image Moderates the Effects of Chronotype on the Risk to Develop an Eating Disorder

**DOI:** 10.3390/jcm14124328

**Published:** 2025-06-17

**Authors:** Anat Lan, Lior Harel, Haim Einat

**Affiliations:** School of Behavioral Sciences, Academic College of Tel Aviv-Yaffo, Tel-Aviv 6818220, Israel; anatla@mta.ac.il (A.L.); liortzimerman1043@gmail.com (L.H.)

**Keywords:** chronotypes, eating disorders, body image

## Abstract

**Background:** Evening chronotypes are increasingly recognized as being at elevated risk of psychological vulnerabilities, including sleep disturbances, mood disorders, and disordered eating. Body image, a central self-related construct, has been identified as a key factor influencing eating disorder (ED) risk. This study aimed to explore whether body image moderates the relationship between chronotype and the risk of developing an ED. **Methods**: A total of 165 adults (139 women; mean age = 35.45) completed validated self-report questionnaires assessing chronotype (Morningness–Eveningness Questionnaire), body image (Body Shape Questionnaire), and ED risk (EAT-26). Pearson correlations and moderation analyses (PROCESS macro and Model 1) were conducted with body image as a moderator of the association between chronotype and ED risk. **Results**: Eveningness was significantly associated with greater ED risk (r = −0.53, *p* < 0.001) and a more negative body image (r = −0.48, *p* < 0.001). Body image was a strong predictor of ED risk (r = 0.85, *p* < 0.001). Moderation analysis revealed a significant interaction between chronotype and body image (b = −0.006, SE = 0.0009, *p* < 0.001), indicating that the relationship between chronotype and ED risk was stronger among individuals with a more negative body image. **Conclusions**: These findings highlight the role of body image as a moderating factor in the link between chronotype and eating disorder vulnerability. Interventions aimed at improving body image, especially among evening chronotypes, may help mitigate ED risk. These results underscore the importance of integrating circadian and psychosocial factors in ED prevention and early intervention strategies.

## 1. Introduction

Individual differences in sleep–wake preferences are termed chronotypes [1]. Although a chronotype has a genetic basis, it is also shaped by external zeitgebers such as exposure to environmental light and the timing and composition of food intake [2]. When not constrained by social obligations, early chronotypes, or morning types (MTs), tend to go to sleep and wake up early, with peak functioning in the morning hours. Late chronotypes, or evening types (ETs), typically prefer later sleep and activity schedules. Individuals whose sleep–wake patterns fall between these two ends of the spectrum are classified as intermediate types [3]. In the general population, morningness–eveningness preferences are distributed along a roughly normal curve [4].

A growing body of evidence indicates that eveningness is associated with poorer sleep quality, more frequent sleep disturbances, greater daytime fatigue, and an elevated risk of mood disorders and depression [5,6]. These associations may, in part, stem from a mismatch between ETs’ biological timing and societal demands, which are typically aligned with morning-oriented schedules. This misalignment, termed social jetlag (SJL), reflects the discrepancy between an individual’s endogenous circadian rhythm and socially imposed activity hours [7].

SJL has been linked to adverse health outcomes, including higher rates of obesity, metabolic syndrome, and disordered eating, as well as greater vulnerability to unhealthy dietary patterns [8,9,10,11,12]. For example, individuals with greater SJL have been found to consume more high-calorie and high-sugar foods, such as sweets and sugary beverages [13,14,15]. A recent systematic review reported that 95% of the 43 studies reviewed found an association between eveningness and at least one unhealthy eating behavior [16]. Such patterns of poor nutrition have been implicated in an increased risk of developing eating disorders (EDs) [17].

Eating disorders are complex psychiatric conditions arising from the interplay of biological, psychological, social, and cultural factors. Core symptoms typically include preoccupation with food and weight, body dissatisfaction, and maladaptive eating behaviors [18]. These disorders can lead to severe medical complications. For instance, individuals with anorexia nervosa face a fivefold increase in premature mortality and a comparable elevation in the risk of chronic illness [19,20]. Other forms of ED, such as bulimia nervosa or binge eating disorder, are associated with complications affecting multiple systems, including gastrointestinal issues due to purging and cardiac dysfunction stemming from electrolyte imbalances [21,22]. The etiology of EDs is heterogeneous, encompassing risk factors such as childhood trauma, psychiatric comorbidities, metabolic disturbances, and negative body image, all of which complicate early detection and prevention [23]. Furthermore, moderation analysis, addressing relationship between several variables concurrently, showed interesting results. Moderation analysis examines whether the strength or direction of the relationship between two variables changes depending on the level of a third variable called the moderator. It helps to identify for whom or under what conditions an effect occurs. Some factors were identified as moderators in previous studies, such as a high BMI, female gender, high social media literacy, body appreciation, and more [24].

Among the key risk factors for EDs is a negative body image, defined as a distorted mental representation of one’s body shape and size [25,26]. These representations are shaped by prior experiences with weight fluctuations, sociocultural norms, personal attitudes, emotional and cognitive traits, and biological predispositions [27]. Internalization of cultural ideals related to thinness or muscularity has been linked to greater body dissatisfaction [28,29]. Such dissatisfaction, as well as fear of weight gain and preoccupation with appearance, are known contributors to ED development [30]. Conversely, a positive body image—characterized by appreciation, acceptance, and respect for one’s body—has been shown to be a protective factor against the risk of developing an ED [31].

The present study aimed to examine the associations between morningness–eveningness preference, body image perception, and the risk of developing an ED. We hypothesized that eveningness would be associated with a more negative body image and a higher risk of developing an ED. Additionally, we hypothesized that body image would moderate the association between chronotype and ED risk, such that a more positive body image would attenuate the strength of this relationship. We chose to explore a non-clinical sample of the general population, as we were mostly interested in the risk factors and sub-clinical traits, behaviors, and attitudes related to body image and disordered eating before they reach diagnostic thresholds. This approach supports early identification, which is crucial in prevention-focused research. Understanding how risk factors (like the evening chronotype) operate in the general population has implications for the development of preventive health strategies and public awareness campaigns.

Importantly, in the current study ED risk was assessed using a self-report questionnaire, reflecting vulnerability rather than a formal clinical diagnosis.

## 2. Materials and Methods

### 2.1. Participants

A convenience sample was reached through social media. The sample included a total of 205 participants, aged 18 and above, who volunteered for this study. Following the exclusion of 40 participants due to incomplete responses, analysis was conducted on a final sample of 165 participants (139 woman, 26 men, mean ± SD for age = 35.45 ± 11.89, age range: 20–67). All procedures were approved by the Institutional Ethics Committee of the Academic College of Tel Aviv-Yaffo (Tel-Aviv, Israel, protocol number 173), and all participants signed an informed consent form.

### 2.2. Instruments and Tools

#### 2.2.1. Demographic Questions

Demographic data were collected regarding each participant’s age, gender, weight, height, and eating disorder diagnoses.

#### 2.2.2. The Morningness–Eveningness Questionnaire (MEQ)

The MEQ is a subjective questionnaire used to assess individuals’ chronotypes. The MEQ comprises 19 items, including Likert-type and time-based items assessing circadian preferences. Scores range from 16 to 86, with higher scores indicating stronger morning preferences and lower scores indicating stronger evening preferences. The global continuous score can be converted to a three-level categorical scale: evening types (16–41), intermediate types (42–58), and morning types (59–86) [32]. In the current study, we used a Hebrew version of the MEQ, which was previously used in other studies [33,34,35]. The internal consistency of the MEQ in the present study was high (α = 0.91).

#### 2.2.3. The Eating Attitudes Test-26 (EAT-26)

The Eating Attitudes Test-26 (EAT-26) is a validated self-report questionnaire used to assess the risk of developing an eating disorder (ED). The EAT-26 consists of 26 items rated on a 6-point Likert scale from 1 (‘never’) to 6 (‘always’). The components of this questionnaire are based on three main indicators: diet, bulimic behavior and excessive preoccupation with food, and oral control. Coded scores range from 0 to 78, with higher scores indicating a greater risk of developing an ED [36]. This study used the Hebrew version of the EAT-26. The internal consistency of the EAT-26 was found to be high, ranging from 0.86 to 0.9 [37].

#### 2.2.4. Body Shape Questionnaire (BSQ)

The BSQ is a self-report questionnaire designed to assess difficulties in a person’s self-image and perception of body shape. The BSQ comprises 34 items, which are rated on a 6-point Likert scale from 1 (‘never’) to 6 (‘always’). Final scores range from 34 to 204, with higher scores indicating a negative body image and lower scores indicating a positive body image. This study used the Hebrew version of the BSQ, which has high internal consistency (α = 0.97) and corresponds to the reports by the authors of the original questionnaire [38].

### 2.3. Procedure

A total of one hundred sixty-five participants signed an informed consent form and completed all online questionnaires using the Qualtrics platform. The participants filled out the demographic questionnaire, the Morningness–Eveningness Questionnaire (MEQ), the Body Shape Questionnaire (BSQ), and the Eating Attitudes Test-26 questionnaire (EAT-26).

### 2.4. Statistical Analysis

Pearson’s r was used to assess bivariate correlations among the studied variables. Student’s *t*-test and Levene’ test for homogeneity of variance were used to examine the effects of gender on the EAT-26 and BSQ data. The Bonferroni correction for multiple comparisons was applied, and accordingly the significance level was set at *p* ≤ 0.01. We tested the hypothesized moderation model using the PROCESS macro for SPSS version 23 (Model 1), following the procedures outlined by Hayes [39]. Body image was examined as a moderator of the association between chronotype (as measured by the Morningness–Eveningness Questionnaire) and the risk of developing an eating disorder (as measured by the EAT-26). A bootstrapping procedure with 5000 resamples was employed to estimate the confidence intervals of the interaction effect. A 95% bias-corrected confidence interval that did not include zero was interpreted as indicating a significant moderation effect. All continuous variables included in the moderation analysis were mean-centered prior to analysis to reduce multicollinearity and facilitate interpretation of the interaction term. Conditional effects were examined at three levels of the moderator (body image), one standard deviation below the mean, near the mean, and one standard deviation above the mean, as recommended by Hayes [39]. In addition, a one-way analysis of variance (ANOVA) was conducted to examine group differences in ED risk across chronotype categories (morning, intermediate, and evening types), based on established MEQ score cutoffs. Post hoc comparisons were performed using the Scheffe test.

## 3. Results

### 3.1. Descriptive Statistics

The means and standard deviations of all variables are presented in Table 1. The chronotype distribution, as assessed by the MEQ, was as follows: morning types (*n* = 32), intermediate types (*n* = 106), and evening types (*n* = 27). The mean ± the SD for the body image score, measured using the BSQ questionnaire, was 90.92 ± 40.09, and the mean ± the SD for the EAT-26 score was 13.64 ± 17.17. Additionally, smoking habits and the presence of an eating disorder diagnosis were assessed. Among the participants, 115 were non-smokers, 25 were social smokers (up to two cigarettes per day), and 25 were regular smokers. In total, 17 subjects were diagnosed with an eating disorder.

### 3.2. Correlational Analysis and T-Tests

Chronotype (the MEQ score) was negatively correlated with ED risk (r = −0.53, *p* < 0.001), such that eveningness was associated with higher risk. Body image was also associated with ED risk (r = 0.85, *p* < 0.001), with a more negative body image predicting higher ED risk. In addition, chronotype was negatively correlated with body image (r = −0.48, *p* < 0.001), indicating that individuals with a stronger evening preference were more likely to report a negative body image. The BMI was normally distributed (*X*^2^(5) = 8.46, *p* = 0.13) and was not correlated with the MEQ (r = 0.08, *p* = 0.31), body image (r = 0.04, *p* = 0.96), or the risk to develop an ED (r = 0.16, *p* = 0.04 [following the Bonferroni correction for multiple comparisons, the significance level was set at *p* ≤ 0.01]). No differences in the homogeneity of variance were found between the genders in the BSQ and EAT-26 scores. For the BSQ, t(163) = 1.27, *p* = 0.21; Levene’s test: F(1163) = 2.39, *p* = 0.12. For the EAT-26, t(163) = 0.52, *p* = 0.6; Levene’s test: F(1163) = 3.83, *p* = 0.052 [note that, as mentioned in the Materials and Methods Section above, following the correction for multiple comparisons the significance level was set at *p* ≤ 0.01]. As no differences were found between the genders, we pooled the data for men and women.

### 3.3. Moderation Analysis

To examine whether body image moderated the relationship between chronotype and ED risk, a moderation analysis was conducted. The analysis included chronotype as the independent variable, body image as the moderator, and ED risk as the dependent variable. The model revealed significant main effects for both chronotype (b = −0.12, SE = 0.05, *p* = 0.02) and body image (b = 0.28, SE = 0.01, *p* < 0.001), indicating that evening preference and a more negative body image were each independently associated with increased ED risk. Notably, the interaction between chronotype and body image was significant (b = −0.006, SE = 0.0009, *p* < 0.001), supporting the hypothesis that body image moderates the relationship between chronotype and ED risk.

**Figure 1:**
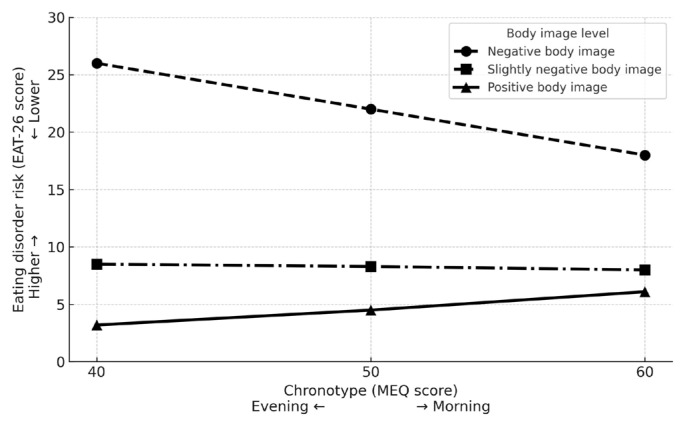
Moderation analysis: the association between chronotype and eating disorder risk as a function of body image.

To probe this interaction, conditional effects were examined at three levels of body image: one standard deviation below the mean or less (reflecting a more positive body image), between one standard deviation below and one standard deviation above the mean, and one standard deviation above the mean or more (reflecting a more negative body image). The results indicated that the association between chronotype and ED risk became stronger as the body image became more negative. Specifically, at high levels of negative body image, chronotype was a strong predictor of ED risk (b = −0.39, *p* < 0.001), whereas this association was not statistically significant at low levels of negative body image (b = 0.13, *p* = 0.06). These results are presented in Figure 1.

### 3.4. Group Differences by Chronotype

**Figure 2:**
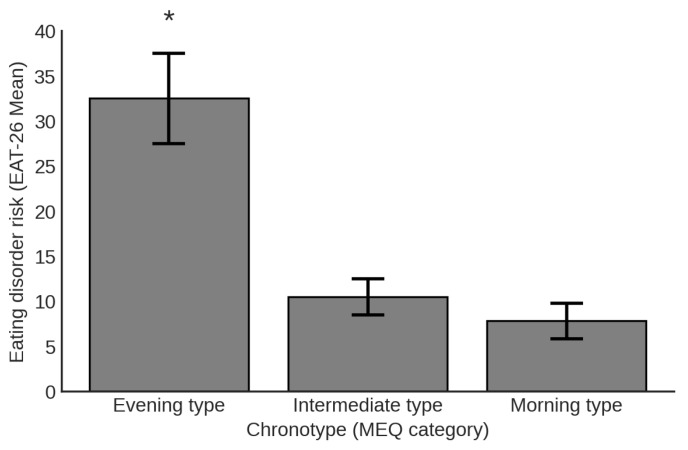
Mean ED risk level (EAT-26 score; higher = greater risk) across chronotype groups (MEQ categories). Error bars represent standard errors of means (SEMs). * Signifies difference from other groups (*p* < 0.001).

To further examine group differences in eating disorder (ED) risk based on chronotype, the MEQ scores were categorized into three groups using established cutoffs: evening types (16–41), intermediate types (42–58), and morning types (59–86). A one-way ANOVA revealed a significant effect of chronotype on ED risk [F(2, 162) = 25.12, *p* < 0.001]. Post hoc comparisons using the Scheffe test indicated that evening types reported significantly higher levels of ED risk compared to both intermediate types (*p* < 0.001) and morning types (*p* < 0.001). No significant difference was found between intermediate and morning types. The magnitude of the difference between evening and morning types was very large (Cohen’s d = 1.53). These differences are illustrated in Figure 2.

### 3.5. Potential Covariates

We examined several potential covariates that could be associated with eating disorder (ED) risk. Age was negatively correlated with ED risk (r = −0.19, *p* = 0.01) and positively correlated with chronotype (r = 0.24, *p* < 0.01). However, both associations were relatively weak. Therefore, age was not included as a covariate in the main analyses. Similarly, the body mass index (BMI) showed a weak negative correlation with ED risk (r = −0.16, *p* < 0.05). Gender and smoking status were not significantly associated with ED risk [gender: t(164) = −0.50, *p* = 0.60; smoking: F(2, 162) = 1.23, *p* = 0.29].

## 4. Discussion

The present study investigated the relationship between chronotype, body image, and the risk of developing an eating disorder (ED), with a specific focus on the moderating role of body image. As hypothesized, individuals with a stronger evening preference were more likely to exhibit higher levels of ED risk and more negative perceptions of their bodies. Moreover, body image significantly moderated the relationship between chronotype and ED risk: the association between eveningness and ED risk was strongest among individuals with a more negative body image.

These findings align with accumulating evidence linking eveningness to greater psychological vulnerability, including poorer sleep quality, social jetlag, mood disturbances, and unhealthy dietary habits [8,10,11]. Studies have specifically demonstrated that evening types are more prone to binge eating, emotional eating, and symptoms of depression compared to their morning-type counterparts [40,41]. These behavioral and emotional patterns may contribute to disordered eating tendencies among evening chronotypes. The relevance of emotional patterns to eating disorders has been demonstrated in many studies. For example, a recent study shows the possible roles of guilt in ED symptomatology [42].

Importantly, the current study adds to the literature by showing that not all individuals with an evening chronotype are equally susceptible to elevated eating disorder risk. Body image played a significant moderating role: those with a more negative body image were particularly vulnerable, while those with a more positive body image appeared to be more resilient. These findings highlight the relevance of self-related psychological factors in shaping vulnerability. Body image is increasingly recognized as a central component of an individual’s self-concept and psychological resilience [43,44], contributing to self-esteem, well-being, and adaptive coping [45,46,47]. As such, a positive body image may serve as a protective factor that mitigates the adverse effects associated with chronotype misalignment.

The observed small but significant negative correlation between the BMI and ED risk warrants further discussion. While this finding is not commonly reported, previous studies have shown inconsistent results regarding the relationship between BMI and disordered eating. Several studies have found positive associations, particularly in the context of binge eating [48,49,50,51], while others reported no significant correlations [52,53]. These discrepancies suggest that the association between the BMI and ED risk may vary across populations, types of eating disorders, and underlying psychological factors.

While we report some interesting results, several limitations of this study must be acknowledged. First, this study’s cross-sectional design limits causal interpretations. Second, the use of self-report measures introduces potential biases related to recall, self-perception, and social desirability. Third, although both women and men were included, the sample was predominantly women, limiting generalizability. The imbalance between women and men may be of special importance because of the higher rates of ED reported in women compared with men [54]. Nonetheless, no differences were found between men and women in the core variables, the BSQ and EAT-26, and the moderation effect remained significant even when the men and women were analyzed separately (Supplementary Table S1), justifying the use of a combined analysis. Finally, recruitment through social media introduces several biases, including the well-documented selection bias where individuals who choose to participate may be different from those who do not. Specifically, it is possible that individuals with a lower body image or a higher risk to develop ED may prefer not to enroll in such studies. However, despite the possible bias, we suggest that the results are valid, at least for this cohort, although they cannot be generalized to all populations.

Furthermore, while the current model focuses on body image, other relevant moderators may also influence the relationship between chronotype and ED risk. Variables such as physical activity and sleep quality [55], stress levels [56], and circadian disruption due to shift work [57] may further shape these associations. Future studies should explore these variables in order to develop a more comprehensive understanding of the multifactorial dynamics underlying eating disorders.

From a clinical perspective, the findings suggest that interventions aimed at improving body image, particularly among evening types, may help buffer against the development of disordered eating. Moreover, behavioral strategies such as sleep education, cognitive behavioral therapy for insomnia (CBT-I), and bright light therapy can be utilized. Such strategies may assist evening chronotypes in aligning their biological rhythms with daily demands, potentially reducing ED vulnerability.

## 5. Conclusions

This study highlights the complex interplay between chronotype, body image, and the risk of developing an eating disorder. The findings suggest that individuals with an evening chronotype may be at increased risk of disordered eating, particularly when coupled with a negative body image. Body image emerged as a significant moderating factor, amplifying the association between eveningness and ED risk. These results underscore the importance of incorporating both circadian and psychosocial dimensions into assessments of ED vulnerability. Interventions that address body image concerns and promote circadian alignment, especially among evening chronotypes, may offer promising avenues for prevention and early intervention.

## Figures and Tables

**Table 1:** Means and standard deviations of all variables.

Variable Name	Mean ± SD
Age	35.45 ± 11.89
Weight	66.07 ± 13.58
Height	166.43 ± 8.64
BMI	23.8 ± 4.0
Chronotype [MEQ score; evening types (16–41), intermediate types (42–58), and morning types (59–86)]	49.22 ± 12.12
Body image (BSQ score; scores range from 34 to 204, with higher scores indicating a negative body image.)	90.92 ± 40.09
Risk of developing an ED (EAT-26 score; scores range from 0 to 78, with higher scores indicating greater risk.)	13.64 ± 17.17

## Data Availability

Raw data is available upon request from the authors.

## Supplementary Materials

The following supporting information can be downloaded at: https://www.mdpi.com/article/10.3390/jcm14124328/s1, Table S1: Moderation analysis predicting eating disorder (ED) risk (EAT-26) from chronotype (MEQ), body image (BSQ), and their interaction, separately for males and females.

## Abbreviations

The following abbreviation is used in this manuscript:

ED	Eating disorder
