# Supplementary material for: Light and Food: Body Image Moderates the Effects of Chronotype on the Risk to Develop an Eating Disorder
Source: J Clin Med. 2025 Jun 17;14(12):4328. doi: 10.3390/jcm14124328 (PMC12194677; doi:10.3390/jcm14124328)
Supplement: Supplementary file 1 [file jcm-14-04328-s001.zip › jcm-3626582-supplementary.pdf]

**Supplementary Table S1**

Moderation analysis predicting eating disorder (ED) risk (EAT-26) from chronotype (MEQ), body image (BSQ), and their interaction, separately for males and females. All continuous variables were mean-centered prior to analysis. The moderation effect remained significant in both groups.

| Variable | B (Female) | SE (Female) | p (Female) | B (Male) | SE (Male) | p (Male) |
|---|---|---|---|---|---|---|
| Intercept | 12.02 | 0.68 | <0 .001 | 12.07 | 1.80 | < 0.001 |
| MEQ (centered) | -0.12 | 0.07 | 0.081 | -0.10 | 0.12 | 0.422 |
| BSQ (centered) | 0.29 | 0.02 | < 0.001 | 0.29 | 0.06 | < 0.001 |
| MEQ × BSQ | -0.0067 | 0.0011 | < 0.001 | -0.0067 | 0.0023 | 0.008 |
